# New records of *Celoporthe guangdongensis* and *Cytospora rhizophorae* on mangrove apple in China

**DOI:** 10.3897/BDJ.8.e55251

**Published:** 2020-11-03

**Authors:** Long yan Tian, Jin zhu Xu, Dan yang Zhao, Hua long Qiu, Hua Yang, Chang sheng Qin

**Affiliations:** 1 Guangdong Academy of Forestry, Guangzhou, China Guangdong Academy of Forestry Guangzhou China

**Keywords:** canker, Diaporthales, pathogen, taxonomy

## Abstract

**Background:**

*Sonneratia apetala* Francis Buchanan-Hamilton (Sonneratiaceae, Myrtales), is a woody species with high adaptability and seed production capacity. *S. apetala* is widely cultivated worldwide as the main species for mangrove construction. However, the study of diseases affecting *S. apetala* is limitted, with only a few fungal pathogens being recorded. Cryphonectriaceae (Diaporthales) species are the main pathogens of plants. They can cause canker diseases to several trees and thereby seriously threaten the health of the hosts. These pathogens include *Cryphonectria parasitica* (Cryphonectriaceae) causing chestnut blight on *Castanea* (Rigling and Prospero 2017) and *Cytospora chrysosperma* (Cytosporaceae) causing polar and willow canker to *Populus* and *Salix* (Wang et al. 2015) . Therefore, the timely detection of of Cryphonectriaceae canker pathogens on *S. apetala* is extremely important for protecting the mangrove forests.

**New information:**

Two diaporthalean fungi, *Celoporthe guangdongensis* and *Cytospora rhizophorae* have been reported for the first time to cause canker on the branches of *S. apetala*. *C. guangdongensis* is significantly pathogenic and *C. rhizophorae* is saprophytic on *S. apetala*.

## Introduction

Mangrove apple (*Sonneratia apetala* Francis Buch.-Ham., Sonneratiaceae, Myrtales), which is the main species of mangrove forests, was introduced to China for restoration purposes in 1985 and its plantation has greatly improved the soil fertility with multitudes of useful features as a pioneer restoration species (Jayatissa et al. 2002, Ren et al. 2009). *S. apetala* has thereby become an important woody species with great economic and ecological importance in China (Ren et al. 2009). However, studies on fungal diseases of *S. apetala* are limited, with only 4 fungal species having been reported as pathogens of this plant so far (namely *Helicascus kanaloanus*, *Lulworthia grandispora*, *Neofusicoccum mangiferae* and *Phomopsis sonneratiae*), which had severely hindered any developmental measures toward the protection of mangrove forests (Farr and Rossman 2019, Qiu et al. 2018).

Species of Cryphonectriaceae Gryzenh. & M.J. Wingf. (Diaporthales), as a group of important pathogens, have been reported to infect bark beetles and wood (Gryzenhout et al. 2004). *Cryphonectria* species, which are the main members of Cryphonectriaceae, can cause serious canker diseases on chestnut, eucalyptus and oak trees (Rigling and Prospero 2017, Jiang et al. 2019, Jiang et al. 2018b). For example, *Cryphonectria parasitica*, *Cryphonectria neoparasitica* and *Cryphonectria japonica* cause chestnut blight on *Castanea* (Jiang et al. 2019); *Cryphonectria cubensis* causes severe stem cankers on *Eucalyptus* (Sharma et al. 2010); *Cryphonectria quercicola* and *Cryphonectria quercus* cause stem canker on *Quercus* (Jiang et al. 2018b). In addition, *Celoporthe* (Cryphonectriaceae, Diaporthales) is a notorious pathogenic genus that infects the barks of Myrtales plants (Ali et al. 2018, Wang et al. 2018). Surveys in southern China for pathogens of trees belonging to the family Myrtaceae identified several *Celoporthe* species (Zhou et al. 2008). *Cytospora* (Cytosporaceae, Diaporthales) is a genus that causes serious dieback and stem canker diseases that commonly affects woody plants (Fan et al. 2015, Wang et al. 2014). These pathogens includes *Cytospora chrysosperma* (Cytosporaceae), which causes polar and willow canker on *Populus* and *Salix* (Fan et al. 2019, Wang et al. 2015). In summary, fungal species of Diaporthales can seriously threaten the healthy growth of several woody plants.

**Figure 1.**
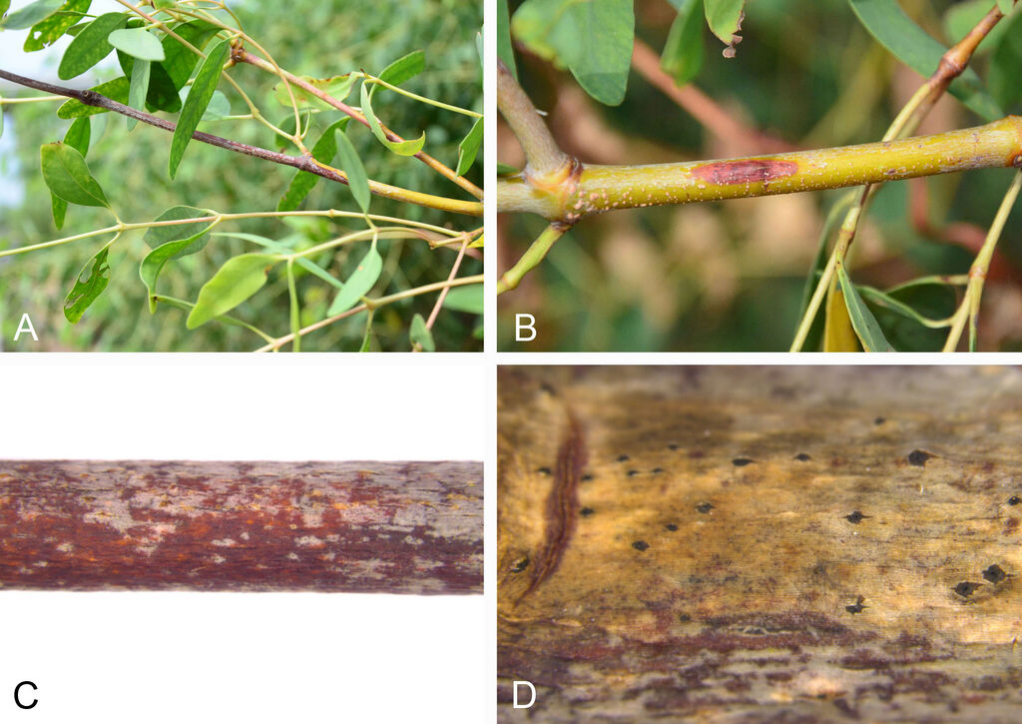
Disease symptoms. **A** and **B.** cankered branches; **C.** Conidiomata of *Celoporthe guangdongensis* on branches; **D.** Conidiomata of *Cytospora rhizophorae* on branches.

Overall, fungal species of the Diaporthales can seriously threaten the healthy growth of mangrove forest when found to infect woody species such as Mangrove apple. During our disease surveys on Mangrove apple trees in Guangdong province, necrosis and canker on the trunks, branches or twigs of *S. apetala* were observed and orange-to-red cankers were photographed (Fig. 1). Two diaporthalean fungi were recognized based on the morphological characteristics of conidiomata and conidia from cankered tissues. These findings can provide significant information toward the protection of Mangrove apple trees, including resistance breeding.

## Materials and methods

### Samples and isolates

The branches of 3 *S. apetala* trees with canker lesions with conidiomata were collected and isolations were conducted in the laboratory for 2 types of infections. For cankered lesions on the bark, the branches were cleaned with tap water and small pieces of bark (sized approximately 2 mm × 2 mm) were cut from the junction of the diseased and healthy portions. These small pieces were disinfected in 75% ethanol for 5 s and transferred to a 3% sodium hypochlorite (NaClO) solution for 2 min. Then, the samples were washed thrice with sterile water and inoculated on the surface of potato dextrose agar (PDA) plates. For branches with conidiomata, single conidial isolates were obtained by removing the spore masses into axenic water in order to obtain the suspension and spread the suspension on to the surface of PDA plates for isolation (Jiang et al. 2018b).

### Morphological studies

The morphological features of the pathogenic fungi were observed on diseased plant tissues following Fan et al. (2018). Species identification was performed based on the morphological characters of the sporocarp produced on the diseased spots. First, cross-sections were made using a double-edge blade. Then, the morphological characters of sporocarp were recorded. For example, the size of conidiomata and locules were measured by using a dissecting stereomicroscope, while the shape and size of conidiophores and conidia were determined using a Leica compound microscope (LM, DM 2500). Finally, sporocarps were sectioned using a hand-operated blade and more than 50 spores were selected randomly and measured under the Leica compound microscope.

### DNA extraction, PCR amplification and sequencing

Aerial mycelium of fungi grown on PDA (for 7 days at 25ºC) was used to extract the genomic DNA. The DNA extraction was performed by the modified CTAB method (Doyle and Doyle 1990). The internal transcribed spacer of rDNA (ITS) was amplified with the primers ITS1 and ITS4 (White et al. 1990). The translation elongation factor-1 alpha (TEF) was amplified with the primers EF1-688F and EF1-1251R (Alves et al. 2008). Two regions within the β-tubulin (BT1/BT2) gene were amplified with the primers Bt1a/Bt1b and Bt2a/Bt2b (Glass and Donaldson 1995). PCR amplification and sequencing were performed following the protocol of Wang et al. (2018). The PCR amplification products were estimated visually by electrophoresis in 2% agarose gel at 60 V for 90 min. DNA sequencing was performed using an ABI PRISM® 3730XL DNA Analyzer with the BigDye® Terminater Kit v.3.1 (Invitrogen) at the Shanghai Invitrogen Biological Technology Company Limited (Beijing, China). Two isolates were detected for each species.

### DNA sequence analysis

The 10 new sequences generated in this study and the reference sequences of *Celoporthe* and *Cytospora* isolates selected from recent studies, were included in the phylogenetic analyses (TW). These sequences were aligned with MAFFT v.7 (Katoh and Toh 2010) and manually adjusted. For *Celoporthe*, phylogenetic analyses were performed, based on the combined ITS-BT1-BT2-TEF sequences by PAUP v. 4.0b10 (Swofford and Sullivan 2003) for Maximum Parsimony (MP), PhyML v.3.0 (Guindon et al. 2010) for Maximum Likelihood (ML) and MrBayes v.3.1.2 (Ronquist and Huelsenbeck 2003) for Bayesian Inference (BI), respectively. For *Cytospora*, ITS sequences were used to conduct phylogenetic analyses using the same software. Information on the isolates, the genes sequenced and GenBank accessions used in this study are all included in Suppl. material 1.

### Pathogenicity analysis

For pathogenicity trials, the 2 isolates TLY1-15 (*Celoporthe guangdongensis*) and TLY2-42 (*Cytospora rhizophorae*) were randomly selected for the inoculation studies. The inoculations were performed on the branches of healthy *S. apetala* trees as per the the methods described by Chen et al. (2011). Briefly, the detached branches (aged: 1–2-years-old, approximately 1.0-cm diameter) from healthy *S. apetala* trees were selected and cut into 20-cm-long pieces. A total of 90 fresh branches were used for the pathogenicity tests and 45 branches were cut and inoculated with each of the two isolates or sterile PDA, respectively. The other branches were scalded and inoculated with the same 2 isolates or sterile PDA. The inoculated branches were sealed in Petri dishes with a gauze immersed in sterile water and maintained in a greenhouse at 25°C.

After 2 weeks, some symptoms were detected on the surface of the inoculated branches. The lesion sizes in the cambium were measured from all experimental and control groups. Re-isolations were performed on PDA and the re-isolation cultures were identified by DNA testing.

Differences in the lesion sizes between the isolates and negative controls were analyzed by one-way analysis of variance (ANOVA), followed by least significant difference (LSD) tests. Statistical analysis was performed by using the R v.3.4.3 software and considered to be significant at P < 0.05.

## Taxon treatments

### Celoporthe guangdongensis

S. F. Chen, Gryzenhout, J. Roux, Y. J. Xie, M.j. Wingfield, & X.D. Zhou (2011)

6F829486-13BC-5D5D-A931-C1F71AB16B0D

#### Materials

- **Type status:** Other material. **Occurrence:** catalogNumber: TLY1-15; TLY1-18; recordedBy: C.S. Qin & L.Y. Tian; **Taxon:** scientificName: Celoporthe guangdongensis; kingdom: Fungi; order: Diaporthales; family: Cytosporaceae; genus: Celoporthe; **Location:** country: China; stateProvince: Guangdong; locality: Zhongshan City, Hengmen village, 113.5810°N, 22.4820°E; verbatimLocality: 2.546 m; **Identification:** identifiedBy: L.Y. Tian; identificationReferences: (Kohlmeyer and Kohlmeyer 1971); **Event:** year: 2018; month: September; day: 26; habitat: on branches of *Sonneratia apetala* Buch.-Ham.; **Record Level:** language: en

#### Description

**Figure 2.**
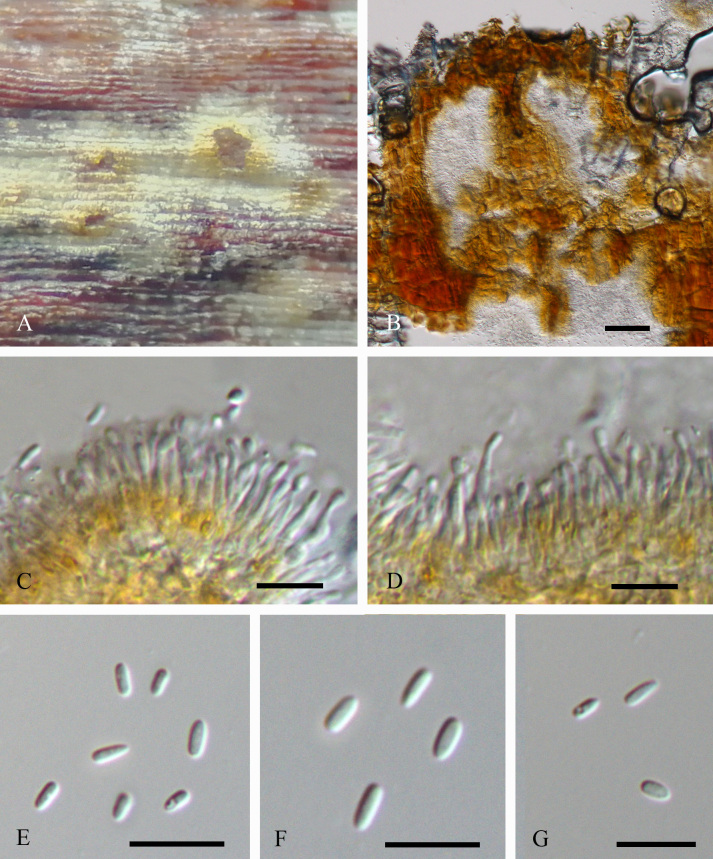
Morphology of *Celoporthe guangdongensis* from *Sonneratia apetala*. **A.** Conidiomata; **B.** Transverse sections through conidiomata; **C–D.** Conidiophores; **E–G.** Conidia. Scale bars: B = 100 μm; C–G = 10 μm.

Conidiomata eustromatic, superﬁcial to slightly immersed, pulvinate to conical without necks, occasionally with a neck, orange when young, black when mature, conidiomatal bases above the bark surface 300–500 µm high, 200–1000 µm diam. Conidiomatal locules with even to convoluted inner surfaces, occasionally multilocular, locules 100–650 µm diam. Stromatic tissue pseudoparenchymatous. Conidiomatal locules multilocular, seldom unilocular, locules 30–500 mm. Conidiophores hyaline, branched irregularly at the base or above, with or without separating septa, (5–)8.5–13.5(–16) × 1.5–2.5 µm. Conidiogenous cells phialidic, determinate, apical or lateral on branches beneath a septum, cylindrical with or without attenuated apices, (1.5–)2–3 µm wide, collarette and periclinal thickening inconspicuous. Conidia hyaline, non-septate, oblong to cylindrical to ovoid, occasionally allantoid, (2.3–)3.1–3.5(–4.6) × (1–)1.5(–2) µm, exuded as bright luteous tendrils or droplets (Fig. 2).

#### Notes

**Figure 3.**
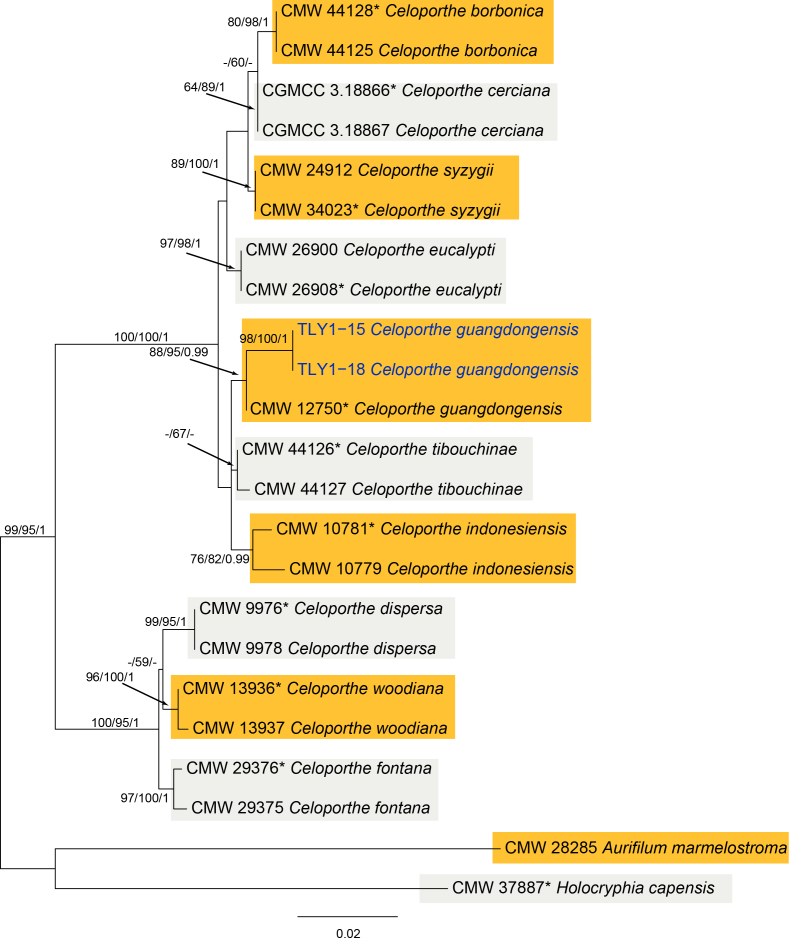
Phylogram of *Celoporthe* from a Maximum Likelihood analysis, based on combined ITS, BT1, BT2 and TEF genes. MP, ML and BI bootstrap support values are shown in order. The tree is rooted with *Aurifilum marmelostroma* and *Holocryphia capensis*. Strains in the current study are in blue.

*Celoporthe guangdongensis* was initially reported on *Eucalyptus* in Guangdong Province of China as a canker pathogen (Chen et al. 2011). Two isolates from the present study cluster in a clade closely related to CMW 12750 (Fig. 3) and conidial dimensions measured in the present study fit exactly with those in Chen et al. (2011).

### Cytospora rhizophorae

Kohlm. & E. Kohlm (1971)

80E71A52-74B4-5FD5-A5AE-AE1E3A00CC3E

#### Materials

- **Type status:** Other material. **Occurrence:** catalogNumber: TLY1-13; TLY2-42; recordedBy: C.S. Qin & L.Y. Tian; **Taxon:** scientificName: Cytospora rhizophorae; kingdom: Fungi; order: Diaporthales; family: Cytosporaceae; genus: Cytospora; **Location:** country: China; stateProvince: Guangdong; locality: Zhongshan City, Hengmen village, 113.5810°N, 22.4820°E; verbatimElevation: 2.546 m; **Identification:** identifiedBy: L.Y. Tian; identificationReferences: (Kohlmeyer and Kohlmeyer 1971); **Event:** year: 2018; month: September; day: 26; habitat: on the twig of *Sonneratia apetala* Buch.-Ham.; **Record Level:** language: en

#### Description

**Figure 4.**
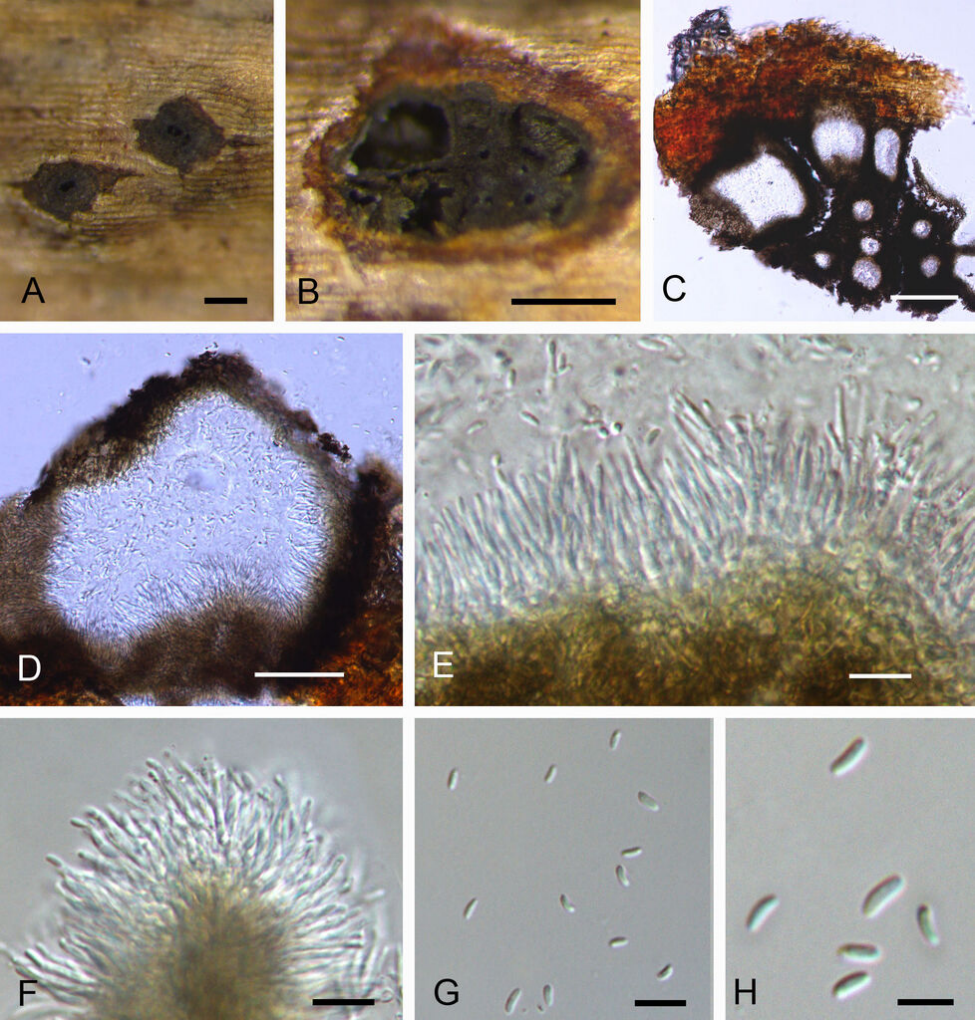
Morphology of *Cytospora rhizophorae* from *Sonneratia apetala*. **A–B.** Conidiomata; **C–D.** Transverse sections through conidiomata; **E–F.** Conidiophores; **G–H.** Conidia. Scale bars: A–B = 200 μm; C = 100 μm; D = 50 μm; E–G = 10 μm; H = 5 μm.

Pycnidial stromata ostiolated, immersed in bark, scattered, erumpent through the surface of bark, discoid, with favaginous multiple locules. Ectostromatic disc black, circular to ovoid, (300–)400–850(–950) µm in diam. Locule numerous, arranged irregularly with common walls, (100–)200–250(–350) µm in diam. Conidiophores hyaline, branched at base or not branched, thin walled, filamentous, (4.5–)6–14(–16) × 1–2 µm. Conidiogenous cells enteroblastic polyphialidic, (1.3–)2–4.5(–5.5) × 1–2.5 µm. Conidia hyaline, allantoid, smooth, aseptate, thin-walled, (3–)3.5–5(–6.1) × 1–1.5 μm (Fig. 4).

#### Notes

**Figure 5.**
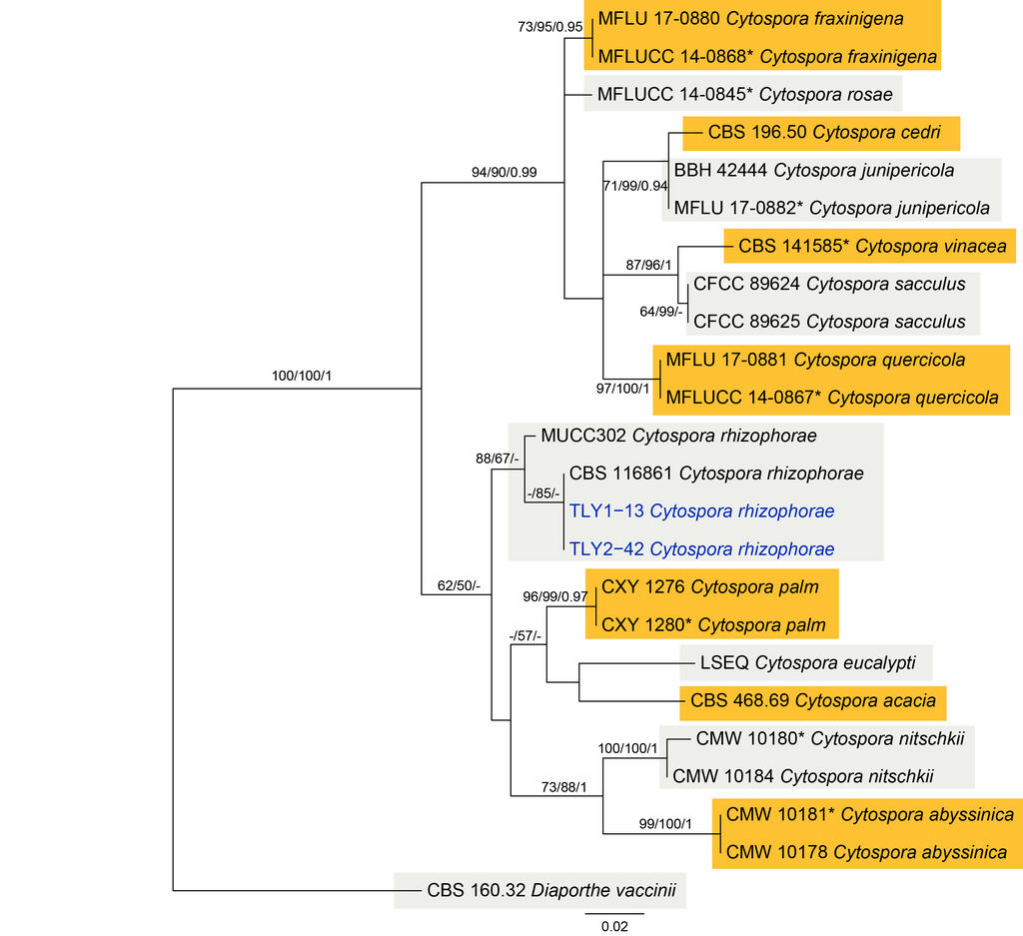
Phylogram of *Cytospora* from a Maximum Likelihood analysis, based on ITS sequences. MP, ML and BI bootstrap support values are shown in order. The tree is rooted with *Diaporthe vaccinii*. Strains in the current study are in blue.

*Cytospora rhizophorae* was initially introduced as mangrove fungi on *Rhizophora* species (Kohlmeyer and Kohlmeyer 1971). Two isolates from the present study, together with MUCC302 and CBS 116861, formed a distinct clade (Fig. 5). Additionally, the morphology observed in this study fit exactly with the primary description (Kohlmeyer and Kohlmeyer 1971).

## Analysis

### Molecular phylogeny

Three isolations (iTLY1-18 inclusive) obtained from the branches of *S. apetala* with canker lesions and 4 isolations (TLY1-15 inclusive) from branches with conidiomata had the same cultural phenotypes on PDA. Another 4 isolations (TLY1-13 and TLY2-42 inclusive) collected from the branches with similar conidiomata showed the same cultural phenotypes on PDA. Finally, we selected the TLY1-18 and TLY1-15, TLY1-13 and TLY2-42 for molecular phylogeny.

In the genus *Celoporthe*, the combined ITS, BT1, BT2 and TEF alignment contained 23 sequences (including 2 outgroups) and 1684 characters including alignment gaps; of which 1387 were parsimony informative, 159 were variable and parsimony uninformative, and 138 were constant. The MP analysis revealed 2 equally most-parsimonious trees; the first tree (TL = 367, CI = 0.907, RI = 0.934, RC = 0.848) is shown in Fig. 3. The topology of the phylogenetic trees obtained from ML and Bayes were similar to that of the MP tree. The *Celoporthe* isolates from the present study cluster in a clade were found to be closely related to the ex-type of *C. guangdongensis* CMW 12750 (Fig. 3).

In the genus *Cytospora*, the ITS alignment contained 24 sequences (including one outgroup) and 620 characters including alignment gaps; of which 442 were parsimony informative, 71 were variable and parsimony uninformative and 107 were constant. The MP analysis resulted in 21 equally most-parsimonious trees; the first tree (TL = 251, CI = 0.853, RI = 0.908, RC = 0.774) is shown in Fig. 5.The topology of phylogenetic trees obtained from ML and Bayes were similar to that of the MP tree. The *Cytospora* isolates from the present study and 2 *C. rhizophorae* strains were clustered in a supported clade (Fig. 5).

### Pathogenicity trials

**Figure 6.**
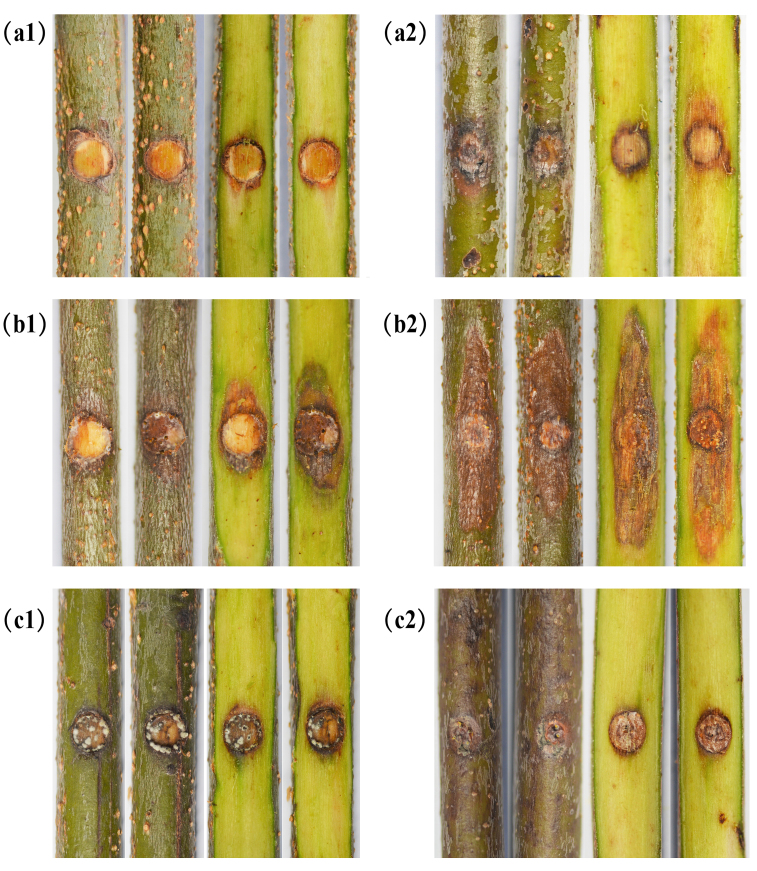
Lesions resulting from inoculation of *Celoporthe guangdongensis* and *Cytospora rhizophorae* on to *Sonneratia apetala* branches and wound response on the negative controls; negative control (a), *Celoporthe guangdongensis* (b), *Cytospora rhizophorae* (c). Line 1, inoculated on to incised wound; line 2, inoculated on to scald wounds.

**Figure 7.**
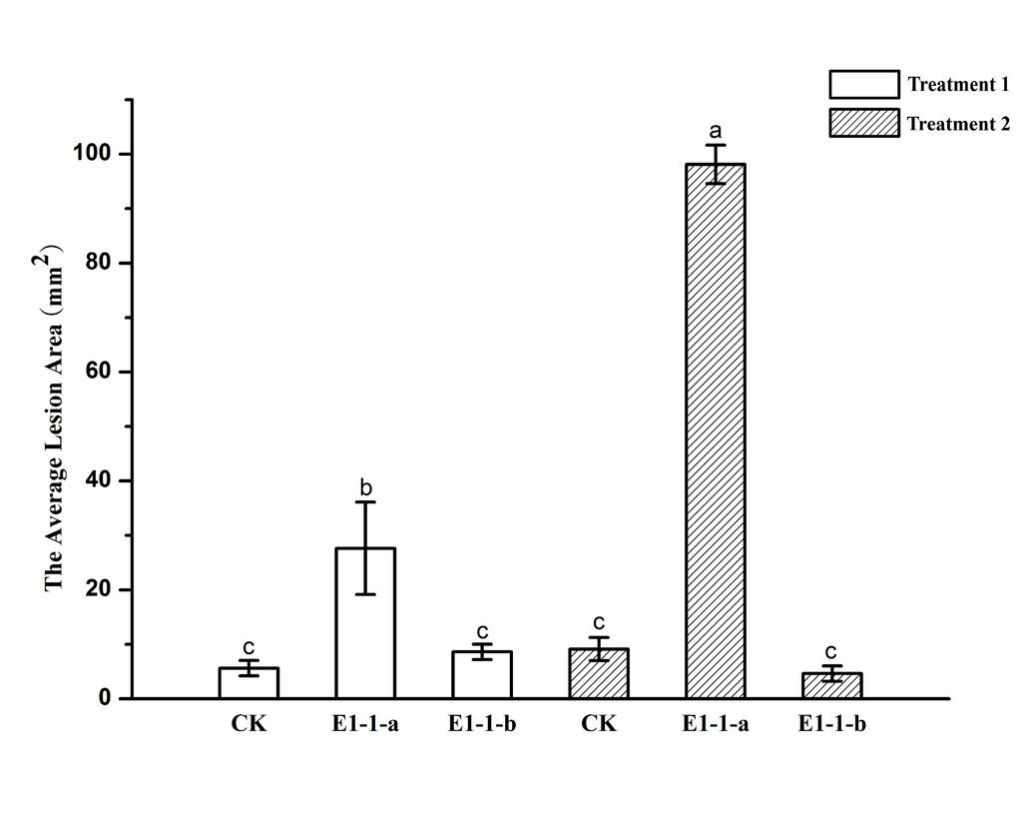
Histogram showing the average lesion area (mm^2^) resulting from inoculations of *Sonneratia apetala* with *Celoporthe guangdongensis* (TLY1-15) and *Cytospora rhizophorae* (TLY2-42). Treatment 1 inoculated on to incised wound; Treatment 2 inoculated on to scald wounds. Bars represent 95% confidence limits for each treatment. Different letters above the bars indicate treatments that statistically were significantly different (P = 0.05).

The isolates of *Celoporthe guangdongensis*, TLY1-15 on *S. apetala* in the greenhouse showed pathogenicity, but no pathogenicity was detected in any of the inoculations with the blank control or *Cytospora rhizophorae* within 6 weeks. Two weeks after inoculation, bark lesion was shown on the scalded branches inoculated with *C. guangdongensis*. Subsequently, the lesion was also exhibited on the wound of *S. apetala* trees branches treated with *C. guangdongensis* (Fig. 6). The two treatments inoculated with *C. guangdongensis* on the branches of *S. apetala* produced significantly longer lesions as compared with that in the control after 4 weeks (P = 0.05) (Fig. 7). Moreover, yellow or orange fruiting structures and cankers were produced on the barks inoculated with *C. guangdongensis* after 4 weeks (Suppl. material 2). However, all treatments inoculated with *C. rhizophorae* and the blank control produced no bark lesions. The cultural phenotypes and ITS sequences of re-isolations were the same as the tested strains.

## Discussion

As important pathogens inhabiting tree barks on several plant hosts, several fungal species belonging to the Diaporthales order have been reported as important fungal taxa in Sordariomycetes (Fan et al. 2018, Jiang et al. 2018a). In the present study, 2 species in Diaporthales, *Celoporthe guangdongensis* and *Cytospora rhizophorae*, were first confirmed on the mangrove plant *S. apetala*, based on the comparisons of their conidial characteristics and DNA sequences data. As reported previously, *Celoporthe* and *Cytospora* are both genera that include several species causing serious economic losses on wood production (Fan et al. 2015, Wang et al. 2018), which suggests that the 2 fungal species reported in this paper mayt severely damage *S. apetala*.

*C. guangdongensis* have been confirmed to be an important canker pathogen on *Eucalyptus* (Myrtaceae, Myrtales), although only 1 isolate has been preserved (Chen et al. 2011). In our study, *C. guangdongensis* isolates were obtained from *S. apetala* (Sonneratiaceae, Myrtales), indicating that *Eucalyptus* trees were not unique host of this species. As *S. apetala* belongs to Myrtales, the conclusion that *Celoporthe* species are host-specific to Myrtales species is accurate based on our research (Chen et al. 2011). Moreover, *C. guangdongensis* arise from both non-native Myrtales species in China, implying that *C. guangdongensis* might not be native to China. However, data on more number of strains from different places are warranted to confirm the origin of *C. guangdongensis*. In addition, considering that *Celoporthe* spp. can infect several plant species (Nakabonge et al. 2006, Wang et al. 2018), it is believe that *C. guangdongensis* may also possess the ability to infect other tree species. Therefore, more research are warranted be design control measures for related diseases.

*C. rhizophorae* has been reported as an endophytic and pathogenic fungus that is host-specific to mangrove plants and occurrs in almost mangrove habitats (Kohlmeyer and Kohlmeyer 1971, Wier et al. 2000). Similarly, *C. rhizophorae* was found on the branches of *S. apetala* in our study. However, this fungus showed no pathogenicity to *S. apetala*, because no extensions were detected on the scalded branches in 6 weeks after innoculation with this strain. In fact, several fungi in the *Cytospora* genus have been reported as necrotrophic pathogens (Zhou et al. 2020, Su et al. 2018). Thus, based on the previous studies by other researchers and the innoculation outcomes in the present study, we can infer that *C. rhizophorae* is presently saprophytic to *S. apetala*. However, the possibility that *C. rhizophorae* can kill the tissues of *S. apetala* cannot be excluded, because the condition of the inoculation test was different from the natural conditions, moreover, *C. rhizophorae* has been reported to cause death in some plants, including *Rizophora mangle* (Perdomo et al. 2018, Wier et al. 2000).

## Supplementary Material

B865B57E-E90A-5749-A604-836C686DD6A6 10.3897/BDJ.8.e55251.suppl1 Supplementary material 1 Isolates used in this study, the genes sequenced and GenBank accessions Data type Table Brief description Isolates used in this study, the genes sequenced and GenBank accessions File: oo_450216.doc https://binary.pensoft.net/file/450216 Long yan Tian,Chang sheng Qin

933C09CE-8C83-52C4-9FE0-91F8A79D9150 10.3897/BDJ.8.e55251.suppl2 Supplementary material 2 Fruiting structures and cankers were produced on the bark inoculated with *C. guangdongensis* after four weeks. Data type image Brief description Fruiting structures and cankers were produced on the bark inoculated with *C. guangdongensis* after four weeks. File: oo_455046.jpg https://binary.pensoft.net/file/455046 Long yan Tian

XML Treatment for Celoporthe guangdongensis

XML Treatment for Cytospora rhizophorae
