# Supplementary material for: New records of Celoporthe guangdongensis and Cytospora rhizophorae on mangrove apple in China
Source: Biodivers Data J. 2020 Nov 3;8:e55251. doi: 10.3897/BDJ.8.e55251 (PMC7655784; doi:10.3897/BDJ.8.e55251)
Supplement: Supplementary material 1 — Isolates used in this study, the genes sequenced and GenBank accessions [file bdj-08-e55251-s001.doc]

Isolates used in this study, the genes sequenced and GenBank accessions.

| Species | Isolate no. | Host | Location | GenBank accession no. | | | |
| --- | --- | --- | --- | --- | --- | --- | --- |
|  |  |  |  | ITS | BT1 | BT2 | TEF |
| *Aurifilum marmelostroma* | CMW 28285 | *Terminalia mantaly* | Cameroon | FJ882855 | FJ900585 | FJ900590 | NA |
| *Celoporthe cerciana* | CGMCC 3.18867 | *Eucalyptus grandis* | China | MH084349 | MH084379 | MH084409 | MH084439 |
| *Celoporthe cerciana* | CGMCC 3.18866* | *Eucalyptus grandis* | China | MH084352 | MH084382 | MH084412 | MH084440 |
| *Celoporthe dispersa* | CMW 9976* | *Syzygium cordatum* | South Africa | DQ267130 | DQ267136 | DQ267142 | HQ730840 |
| *Celoporthe dispersa* | CMW 9978 | *Syzygium cordatum* | South Africa | AY214316 | DQ267135 | DQ267141 | HQ730841 |
| *Celoporthe eucalypti* | CMW 26900 | *Eucalyptus* sp. | China | HQ730836 | HQ730816 | HQ730826 | HQ730849 |
| *Celoporthe eucalypti* | CMW 26908* | *Eucalyptus* sp. | China | HQ730837 | HQ730817 | HQ730827 | HQ730850 |
| *Celoporthe fontana* | CMW 29376* | *Syzygium guineense* | Zambia | GU726941 | GU726953 | GU726953 | JQ824074 |
| *Celoporthe fontana* | CMW 29375 | *Syzygium guineense* | Zambia | GU726940 | GU726952 | GU726952 | JQ824073 |
| *Celoporthe guangdongensis* | CMW 12750* | *Eucalyptus* sp. | China | HQ730830 | HQ730810 | HQ730820 | HQ730843 |
| ***Celoporthe guangdongensis*** | **TLY1-15** | ***Sonneratia apetala*** | **China** | **MT731586** | **MT740224** | **MT740226** | **MT740228** |
| ***Celoporthe guangdongensis*** | **TLY1-18** | ***Sonneratia apetala*** | **China** | **MT731587** | **MT740225** | **MT740227** | **MT740229** |
| *Celoporthe indonesiensis* | CMW 10781* | *Syzygium aromaticum* | Indonesia | AY084009 | AY084033 | AY084021 | HQ730842 |
| *Celoporthe indonesiensis* | CMW 10779 | *Syzygium aromaticum* | Indonesia | AY084007 | AY084031 | AY084019 | NA |
| *Celoporthe borbonica* | CMW 44128* | *Tibouchina grandiflora* | La Réunion | MG585741 | MG585725 | NA | NA |
| *Celoporthe borbonica* | CMW 44125 | *Tibouchina grandiflora* | La Réunion | MG585740 | MG585724 | NA | NA |
| *Celoporthe syzygii* | CMW 24912 | *Syzygium cumini* | China | HQ730833 | HQ730813 | HQ730823 | HQ730846 |
| *Celoporthe syzygii* | CMW 34023* | *Syzygium cumini* | China | HQ730831 | HQ730811 | HQ730821 | HQ730844 |
| *Celoporthe tibouchineae* | CMW 44126* | *Tibouchina grandiflora* | La Réunion | MG585747 | MG585731 | NA | NA |
| *Celoporthe tibouchineae* | CMW 44127 | *Tibouchina grandiflora* | La Réunion | MG585748 | MG585732 | NA | NA |
| *Celoporthe woodiana* | CMW 13936* | *Tibouchina granulosa* | South Africa | DQ267131 | DQ267137 | DQ267143 | JQ824071 |
| *Celoporthe woodiana* | CMW 13937 | *Tibouchina granulosa* | South Africa | DQ267132 | DQ267138 | DQ267144 | JQ824072 |
| *Cytospora abyssinica* | CMW 10181* | *Eucalyptus globulus* | Ethiopia | AY347353 | NA | NA | NA |
| *Cytospora abyssinica* | CMW 10178 | *Eucalyptus globulus* | Ethiopia | AY347354 | NA | NA | NA |
| *Cytospora acaciae* | CBS 468.69 | *Ceratonia siliqua fruit* | Spain | DQ243804 | NA | NA | NA |
| *Cytospora cedri* | CBS 196.50 | NA | Italy | AF192311 | NA | NA | NA |
| *Cytospora eucalypti* | LSEQ | *Sequoia sempervirens* | USA | AY347340 | NA | NA | NA |
| *Cytospora fraxinigena* | MFLU 17-0880 | *Fraxinus ornus* | Italy | MF190134 | NA | NA | NA |
| *Cytospora fraxinigena* | MFLUCC 14-0868* | *Fraxinus ornus* | Italy | MF190133 | NA | NA | NA |
| *Cytospora junipericola* | BBH 42444 | *Juniperus communis* | Italy | MF190126 | NA | NA | MF377579 |
| *Cytospora junipericola* | MFLU 17-0882* | *Juniperus communis* | Italy | MF190125 | NA | NA | MF377580 |
| *Cytospora nitschkii* | CMW 10180* | *Eucalyptus globulus* | Ethiopia | AY347356 | NA | NA | NA |
| *Cytospora nitschkii* | CMW 10184 | *Eucalyptus globulus* | Ethiopia | AY347355 | NA | NA | NA |
| *Cytospora palm* | CXY 1276 | *Cotinus coggygria* | China | JN402990 | NA | NA | KJ781296 |
| *Cytospora palm* | CXY 1280* | *Cotinus coggygria* | China | JN411939 | NA | NA | KJ781297 |
| *Cytospora quercicola* | MFLU 17-0881 | *Quercus* sp. | Italy | MF190128 | NA | NA | NA |
| *Cytospora quercicola* | MFLUCC 14-0867* | *Quercus* sp. | Italy | MF190129 | NA | NA | NA |
| *Cytospora rhizophorae* | MUCC302 | *Eucalyptus grandis* | Australia | EU301057 | NA | NA | NA |
| *Cytospora rhizophorae* | CBS 116861 | *Eucalyptus grandis* | Thailand | KY051831 | NA | KX964922 | NA |
| ***Cytospora rhizophorae*** | **TLY1-13** | ***Sonneratia apetala*** | **China** | **MT731588** | **NA** | **NA** | **NA** |
| ***Cytospora rhizophorae*** | **TLY2-42** | ***Sonneratia apetala*** | **China** | **MT731589** | **NA** | **NA** | **NA** |
| *Cytospora rosae* | MFLUCC 14-0845* | *Rosa canina* | Italy | MF190131 | NA | NA | NA |
| *Cytospora sacculus* | CFCC 89624 | *Juglans regia* | China | KR045645 | NA | KR045686 | KP310860 |
| *Cytospora sacculus* | CFCC 89625 | *Juglans regia* | China | KR045646 | NA | KR045687 | KP310861 |
| *Cytospora vinacea* | CBS 141585* | *Vitis interspecific* | USA | KX256256 | NA | KX256235 | KX256277 |
| *Diaporthe vaccinii* | CBS 160.32 | *Vaccinium macrocarpon* | USA | KC343228 | NA | NA | NA |
| *Holocryphia capensis* | CMW 37887* | *Metrosideros angustifolia* | South Africa | JQ862854 | JQ862895 | JQ862936 | JQ863051 |
