# Supplementary figures and images for: New records of Celoporthe guangdongensis and Cytospora rhizophorae on mangrove apple in China
Source: Biodivers Data J. 2020 Nov 3;8:e55251. doi: 10.3897/BDJ.8.e55251 (PMC7655784; doi:10.3897/BDJ.8.e55251)

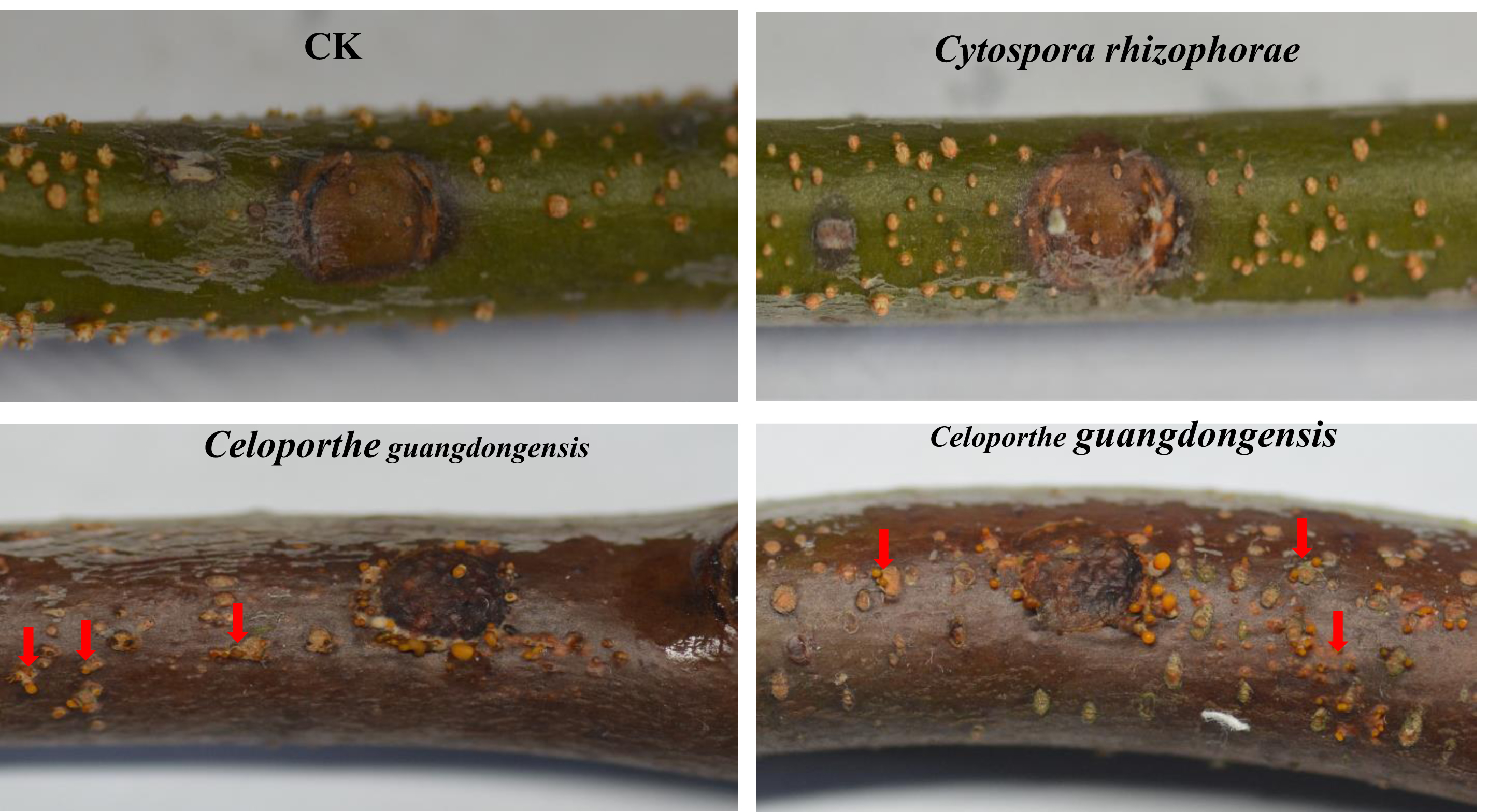

Supplement: Supplementary material 2 — Fruiting structures and cankers were produced on the bark inoculated with C. guangdongensis after four weeks. [file bdj-08-e55251-s002.jpg]
